# A qualitative assessment of barriers and facilitators associated with addressing social determinants of health among members of a health collaborative in the rural Midwest

**DOI:** 10.1186/s12913-021-06859-6

**Published:** 2021-08-24

**Authors:** Kim Nichols Dauner, Lacey Loomer

**Affiliations:** Department of Economics and Health Care Management, Labovitz School of Business & Economics, University of Minnesota Duluth, 1318 Kirby Drive, Duluth, MN 55812 USA

**Keywords:** Rural Health services, Social determinants of Health, Intersectoral collaboration

## Abstract

**Purpose:**

Rural communities have unique economic and social structures, different disease burdens, and a more patchworked healthcare delivery system compared to urban counterparts. Yet research into addressing social determinants of health has focused on larger, urban, integrated health systems. Our study sought to understand capacities, facilitators, and barriers related to addressing social health needs across a collaborative of independent provider organizations in rural Northeastern Minnesota and Northwestern Wisconsin.

**Methods:**

We conducted qualitative, semi-structured interviews with a purposive sample of 37 key informants from collaborative members including 4 stand-alone critical access hospitals, 3 critical access hospitals affiliated with primary care, 1 multi-clinic system, and 1 integrated regional health system.

**Findings:**

Barriers were abundant and occurred at the organizational, community and policy levels. Rural providers described a lack of financial, labor, Internet, and community-based social services resources, a limited capacity to partner with other organizations, and workflows that were less than optimal for addressing SDOH. State Medicaid and other payer policies posed challenges that made it more difficult to use available resources, as did misaligned incentives between partners. While specific payer programs and organizational innovations helped facilitate their work, nothing was systemic. Relationships within the collaborative that allowed sharing of innovations and information were helpful, as was the role leadership played in promoting value-based care.

**Conclusions:**

Policy change is needed to support rural providers in this work. Collaboration among rural health systems should be fostered to develop common protocols, promote value-based care, and offer economies of scale to leverage value-based payment. States can help align incentives and performance metrics across rural health care entities, engage payers in promoting value-based care, and bolster social service capacity.

## Introduction

Rural communities have unique economic and social structures, face different disease burdens, have fewer employers that provide health insurance, and have a more patchworked healthcare delivery system compared to urban areas [1]. Rural health care delivery organizations (HCOs), like their urban counterparts, are increasingly addressing population health in the communities they serve [2] recognizing that factors such as affordable housing, food insecurity, and a lack of transportation, known as social determinants of health (SDOH), can impact their patients’ health outcomes [3].

HCOs have begun to address SDOH in a myriad of ways at the patient-level and the community-level. At the patient-level, HCOs are screening patients for social needs [4] and then integrating assessment findings into the electronic health record (EHR) [5]. Next, HCOs are providing care coordination, patient navigation, social worker, and discharge planning services for patients with social health needs to connect them with community resources [6]. At the community-level, HCOs are conducting Community Health Needs Assessment [7] and engaging in multi-sectoral partnerships to promote health and/or address the underlying systemic issues creating health disparities [6].

Several federal policies and initiatives encourage HCOs to pay attention to population health including Community Health Needs Assessment; value-based payment approaches such as Medicare’s Accountable Care Organizations (ACOs), medical homes, and the Accountable Health Communities model. The Affordable Care Act required tax-exempt hospitals to conduct a Community Health Needs Assessment every 3 years with community stakeholders to assess and formulate a plan that pertains to health care issues within the community they serve. Value-based payment programs promote population health broadly by shifting incentives to focus on outcomes rather than the production of medical services. The Accountable Health Communities model is a pilot program that tests screening for SDOH and integrating community service navigation services within the health care delivery system. Beginning in 2015, with the expansion of disease codes under the 10th revision of International Classification of Diseases, providers can code for SDOH with Z-codes. Expanding codes to include SDOH is an opportunity to document the extent of specific SDOH needs within the health care system; however, they are underutilized [8]. States have also incentivized collaborations between communities and HCOs, including several that specifically help rural communities advance population health [9].

Past research on how HCOs address SDOH has primarily been set in highly integrated settings (e.g. ACOs) [10–14]. Common themes included challenges/facilitators related to community, organization resources, and leadership. Community challenges included location in disadvantaged communities and weak partnering capabilities (e.g. patient data not captured in the EHR), while facilitators included large market share and longstanding community ties [10–12]. Studies have identified clinician buy-in and leadership is important when engaging in SDOH activities but organizational barriers including financial and labor resources (e.g. no direct funding) compromise the ability of HCOs to engage in this work [10, 11]. Summing the field in a systematic review, Gottleib et al. found that HCO-led interventions to address social determinants varied considerably; most have focused on process (e.g. screening/referring), with studies on health, utilization, or costs outcome being fewer in number and having mixed findings (e.g. some reported reductions in emergency department utilization while some report no significant reduction) [13]. Furthermore, although partnership is encouraged by these initiatives and payers, current programs don’t align incentives or outcomes across organizations effectively [14].

Despite the recent growth of research on this topic, there has been little systematic research documenting the scope of activities across diverse HCOs; how HCOs build the capacity to prioritize, develop, and implement such strategies; or what facilitates or challenges that work, especially within organizations not funded by major federal initiatives [15]. Additionally, research into addressing SDOH has focused on larger, urban, integrated health systems. The study sought to fill in some of these gaps in the literature and its purpose is to better understand how various capabilities, facilitators, and barriers impact how social health needs are addressed across rural health care delivery organizations.

## Methods

### Study design and setting

**Fig. 1:**
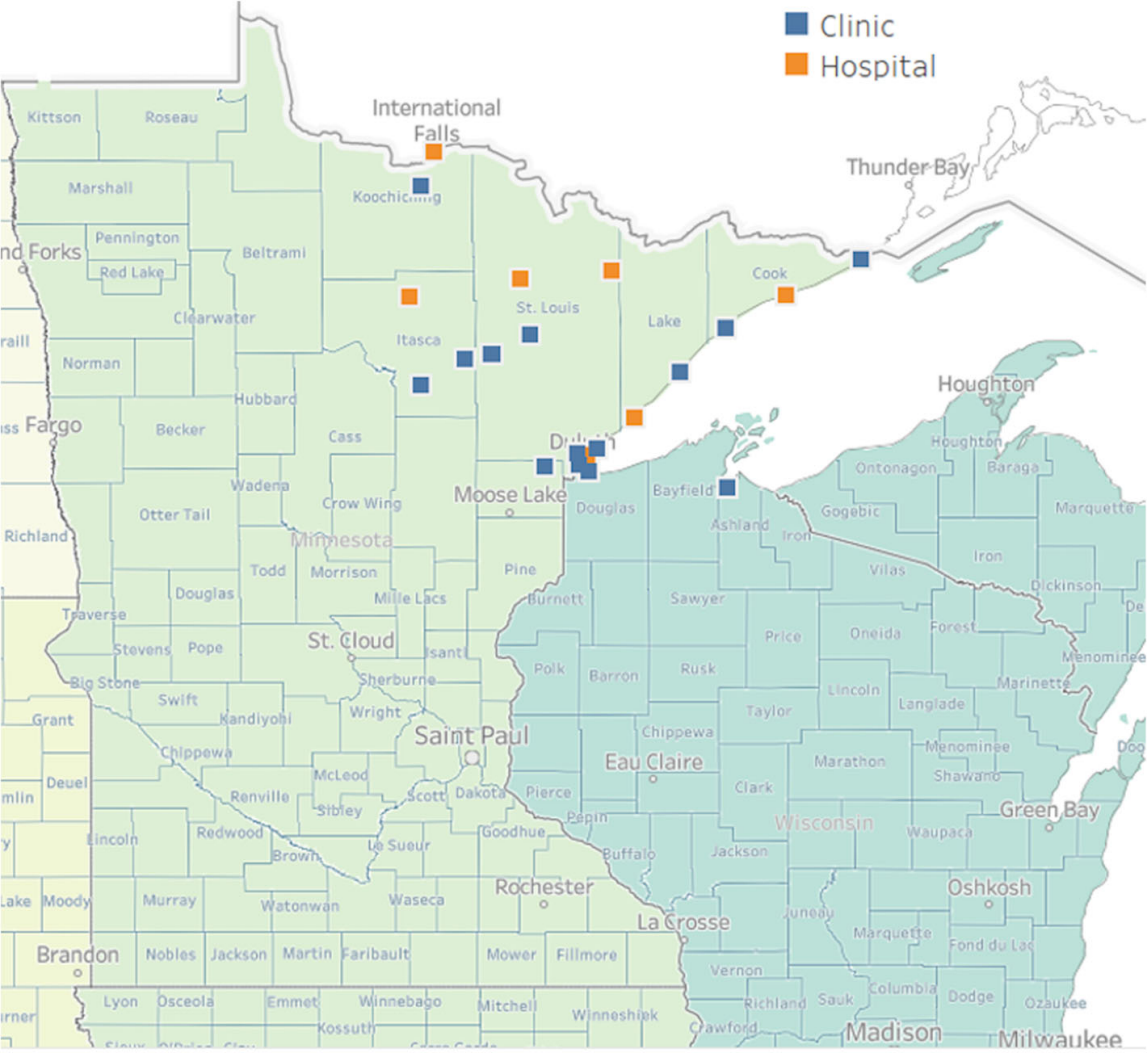
Map of Wilderness Health Members

This research is a case study providing an in-depth, detailed, qualitative, in-situ examination into how a rural health collaborative (Wilderness Health) addresses SDOH. Wilderness Health is a collaborative of 10 independent health care organizations, each of which is independently owned and operated. Although affiliation with Wilderness Health is voluntary, the collaborative itself has both employer-based and Medicaid ACO contracts. Table 1 describes each member organization, which includes four stand-alone critical access hospitals, three critical access hospitals with an affiliated primary care clinic, one multi-clinic system, and one integrated regional health system. Member organizations are located in rural Northeastern Minnesota and Northwestern Wisconsin (Fig. 1). Together, the collaborative members serve an area of approximately 450,000 persons [16].

**Table 1:** Member Organizations of Wilderness Health

Health Care Organization, City, County, State	Description
Bigfork Valley Hospital Bigfork, Itasca County, MN	General medical and surgical hospital with 20 beds, four specialty clinics, pharmacy, and senior services (adult daycare, home care, long term care and independent/assisted living) Government hospital district
Community Memorial Hospital Cloquet, St. Louis County, MN	Critical access, general medical and surgical hospital with 25 beds, 44-bed long term care facility and two clinics Non-government, not-for-profit
Cook Hospital Cook, St. Louis County, MN	Critical access, general medical and surgical hospital with 14 beds and a 28-bed skilled nursing facility Government hospital district
Ely-Bloomenson Community Hospital Ely, St. Louis County, MN	Critical access, general medical and surgical hospital with 21 beds and a pharmacy Non-government, not-for-profit
Fairview Range / Range Regional Health Services Hibbing, St. Louis County, MN	Three primary care clinics, memory care, home care and senior services, inpatient behavioral health unit, pharmacy and general medical and surgical hospital with 81 beds Non-government, not-for-profit
Fairview Health Services / Grand Itasca Clinic and Hospital Grand Rapids, St. Louis County, MN	Multi-specialty clinic with over 60 providers and a general medical and surgical hospital with 50 beds and pharmacy Non-government, not-for-profit
Lakeview Hospital Two Harbors, Lake County, MN	Critical access, general medical and surgical hospital with 17 beds, two primary care clinics (Two Harbors and Silver Bay) and pharmacy Non-government, not-for-profit; part of St. Luke’s health care system
North Shore Health Grand Marais, Cook County, MN	Critical access, general medical and surgical hospital with 16 beds and a 37-bed skilled nursing facility Government hospital district
Rainy Lake Medical Center International Falls, Koochiching County, MN	Critical access, general medical and surgical hospital with 25 beds and one primary care clinic Non-government, not-for-profit
St. Luke’s Duluth, St. Louis County, MN	Health care system with 267 hospital beds, 13 primary care clinics (7 in Duluth, one each in Hermantown, Hibbing, and Mt. Iron, MN, Ashland and Superior WI), 23 specialty clinics, one pharmacy, six urgent care and two express care sites Non-government, not-for-profit

### Qualitative framework

A realist framework, which takes the perspective that actions are grounded in beliefs, reasons, and motivations, shaped our research [17]. This approach was chosen for its usefulness in understanding how something works “on the ground.” As applied to our research specifically, the realist framework was useful for understanding the relationships and interactions between participant’s perspectives on SDOH, their actions at work to address SDOH, and how their perceptions of their organizations and communities impact those actions when it comes to addressing patients’ social health needs in rural communities.

### Research team and relationship to participants

 The first author (KND) is an Associate Professor of Health Care Management at the University of Minnesota Duluth and has over a decade of experience in qualitative methodologies and in evaluating community health collaborations. The second author (LL) is an Assistant Professor of Health Care Management at the University of Minnesota Duluth whose research focuses on rural health care organizations and payer policies. She has experience in qualitative data analysis. KND was asked by Wilderness Health to help their organization better understand how to address social determinants of health across its members. With input from Wilderness Health staff she designed the study and conducted all focus groups and interviews. She was not paid for the work and was viewed by participants as an outside collaborator. Wilderness Health staff and all interview participants have had the opportunity to review and reflect upon the themes identified and provide feedback to the research team.

### Sampling and data collection procedures

Wilderness Health staff and KND generated a list of potential participants, based on their job function within their respective organizations, to interview. Key job functions of interest included direct patient care as related to social health needs (e.g. care coordinators, case managers, primary care physicians), managers of those in direct patient care, and organizational leadership. Because Wilderness Health is a collaborative and members are independently owned and operated, the titles of persons in those roles varied considerably. Wilderness Health staff provided contact information for those persons and sent an introductory email. KND followed up with an email and scheduled interviews with participants. Wilderness Health staff also set up two focus groups, one with care coordinators and case managers on their Medicaid ACO contract and one with the Directors of Nursing from their inpatient facilities. Study procedures were reviewed by the Institutional Review Boards of the University of Minnesota and Wilderness Health and deemed to meet their institutional ethical standards; given that the questions presented less than minimal risk to participants, the need for further review was waived.

Interviews and both focus groups were conducted between July and October 2020. Most were conducted over zoom or phone; one was conducted in person. Interview participants were asked for their consent to participate and be audio recorded. Participants were also informed of the voluntary nature of the study. Each interview lasted between 20 and 65 min. Thirty-seven unique persons were interviewed, including 28 individuals in single and two-person interviews and 15 individuals in two focus groups (six individuals participated in both a focus group and a subsequent interview). Three people did not respond to interview requests (each potential interview respondent was sent two reminders after the initial invite, with each email being approximately 10 days apart). Otter.ai software was used to record and transcribe all interviews electronically. Immediately after the interview, transcripts were reviewed for accuracy, supplemented with the interviewer’s notes, and de-identified to ensure respondent confidentiality.

### Interview questions and guide

An initial set of questions was developed and pilot tested during the two focus groups, which were held first to get a broad perspective. These initial questions were too specific given the variety of job functions and tasks represented across interview participants. As such, questions were revised to be more open ended. This allowed for participants to express the variety of ways they engage across the spectrum of activities related to SDOH and relay the barriers and facilitators they felt most acutely. Probes were used to elicit more information on specific activities, barriers and facilitators and were informed by Chaudoir’s research [18] on structural, organizational, provider, patient, and innovation level factors affecting innovation implementation and by the Greenhalgh Model for Diffusion of Innovations in Service Organizations [19]. The final questions were:

1. What does you/your organization/unit do to address the social health needs of patients?
  *Probes for:* screening activities; documentation in EHR; referral; partnerships with social service organizations, schools, etc.; advocacy efforts.
2. What things help you to do this work?
  *Probes for:* elements of the innovations (e.g. pre-loaded screening tools in EHRs, perceptions of need and relevance, knowledge of how to screen), the organizational structures and systems required for the full implementation (e.g. leadership, integration into workflow, readiness), and external factors (e.g. payment models, partnership quality).
3. What challenges do you face in doing this work?
  *Probes for:* Difficulties arising from elements of the innovations, the organizational structures and systems required for the full implementation, and external factors.
4. What would help you address SDOH/the social needs of patients more effectively?

### Analysis

First, an a priori codebook was developed based on the interview questions. The initial coding was deductive and included tasks associated with SDOH, facilitators, challenges/barriers. Subcoding of tasks included screening, referrals, participation/leadership in community coalitions, partnering with community-based agencies, and internal leadership/communication. The subcoding of challenges and facilitators was more inductive. Subcodes of facilitators included elements of innovation, organizational structure and systems, organizational/innovation processes, external and person factors. Finally, subcodes of challenges included elements of innovation, organizational structure and systems, organizational/innovation processes, and external factors (e.g. community and policy).

After all interviews were completed, both authors separately coded three transcripts, then met and came to consensus on the codes. Then they coded the remaining transcripts, addressed any coding discrepancies, refined and added additional subcodes within the major themes using an iterative process, ultimately coming to consensus on all themes. During analysis, themes seemed to reach a point of saturation, wherein most codes were developed within the initial subset of interviews. Nvivo software version 12 [20] was used for all coding and analysis. The authors also used data from Wilderness Health [21] and the Agency for Health Care Research and Quality database on SDOH [22] to provide contextual information on the communities in which these HCOs serve.

## Results

### Interview participants

Thirty-seven unique persons were interviewed. At least one person was interviewed from each of the 10 organizations comprising Wilderness Health. All interviewees played a key role within their organization’s efforts to address SDOH (see Table 1, above, for more information on each member organization). The largest share of participants (*n* = 11, 30%, including joint appointments) were from St. Luke’s, which reflects its larger size in reference to the other organizations. Job titles represented across the interviews included care coordinators, care navigators, CEOs and VPs, case managers, Clinic Directors, Directors of Nursing, physicians, nurses, social workers, and quality and project managers.

### The community

The counties in which each specific member organization operates vary with regard to their population characteristics and specific needs. However, in general the seven county area has complex social needs (Table 2). The percent of households that received food stamps in the last year ranged from 6% (Cook, MN) to 20% (Ashland, WI). The percent of the county population with means-tested health insurance ranged from 13.0% (Lake, MN) to 24.0% (Koochiching, MN). Most counties had about a quarter of their population that was aged 65 years or older.

**Table 2:** County Characteristics related to SDOH

County Name, State	Cook, MN	Itasca, MN	Koochiching, MN	Lake, MN	St. Louis, MN	Ashland, WI	Douglas, WI
**Population**	5393	45,108	12,440	10,658	199,754	15,600	43,208
**Rural Urban Continuum Code (2013)**	Completely rural or less than 2500 urban population, not adjacent to a metro area	Urban population of 2500 to 19,999, adjacent to a metro area	Urban population of 2500 to 19,999, adjacent to a metro area	Urban population of 2500 to 19,999, adjacent to a metro area	Counties in metro areas of 250,000 to 1 million population	Urban population of 2500 to 19,999, not adjacent to a metro area	Counties in metro areas of 250,000 to 1 million population
**% 65+**	26.6	22.0	23.3	25.1	18.3	18.2	17.5
**Mental health care providers/ 100,000**	92.6	237.1	119.7	66.5	231.0	277.4	115.5
**% Households that received food stamps, past 12 months**	6.4	11.7	15.7	7.5	11.0	20.3	13.9
**% Households with any internet connection**	80.8	76.9	72.9	80.2	77.6	70.9	77.3
**% Means-tested health insurance**	13.1	19.2	24.2	13.0	16.1	23.1	16.6
**% Public Insurance**	22.2	25.8	31.9	19.4	21.6	30.9	22.7
**% of employed in arts, entertainment, recreation, accommodation and food services**	29.8	9.7	9.9	13.0	11.4	11.0	9.7
**% non-civilian pop. Below poverty**	13.6	13.3	17.1	9.3	17.1	13.6	13.1

### Themes

Participants described the ways in which their organizations addressed SDOH, what challenges they face when doing that work, and what facilitates that work. They also discussed the nuances of rural geography and the COVID-19 pandemic and how those two forces shape, challenge and facilitate their work. The themes uncovered overlap due to the complexity of addressing SDOH in a rural HCO, and how the rural geography often exacerbated challenges, but at times enhanced facilitators. We identified themes at different ecological levels: Organization, Community, and External/State and explored barriers, facilitators and activities related to SDOH as subthemes. Interviewees mainly spoke from their vantage point within their respective organizations and thus provided a richer perspective into the various challenges and facilitators. Generally, there was large agreement on the themes across all interview participants. We present themes and illustrative quotes in Table 3.

**Table 3:** Overview of themes

Domain	Subdomain	Illustrative Examples
**Organization**	**Work-flows for Screening and Referral as a Facilitator**	**Physician**: *“I think one barrier would be consistency. And so while you might be able to get one thing at one practice, is that really the same thing that happens across the system, right? And ensuring that consistency. So that when you as the parent go in, are you filling out the same forms and being asked the same questions that you were when you went with your kid … Having that recall. Oh, they’re supposed to be seen a lot so somebody knows that, not just me, but the front desk knows that, so yes, they will definitely get them in in a month, or...it’s a system wide effort to remember all these little gaps, and you know all the little points that need to be filled in, to meet the quality measure...so managing that process and then making sure the person, or somebody is responsible to do the job.”*
	**Work-flows for Screening and Referral as a Barrier**	**Director of Nursing**: *“So our case management team, we have, you know, I suppose, like most facilities have daily rounds where you’re looking at discharge planning and last year one program that we implemented was including our ambulatory care coordinators in the conversation, so that when the patient is discharged from hospital, someone, there’s a warm handoff of information so that somebody is going to be following up.”*
	**Buy-in/Leadership**	**Care Coordinator**: “*I feel like [the organization I work for] has given us the opportunities to do a lot of learning, which I think has helped us. My learning in particular has you know, I, I had had, I don’t know now [but], looking forward after COVID and everything what this will look like in the next year or so, but usually I’ll have like a learning budget to attend conferences and things like that. So that’s valued by [my organization]*.”
**Community**	**Resources**	**Director of Nursing:***“Internet access is somewhat of an issue. Transportation is always [an issue]. No coverage with needed services in rural areas. No long term care beds or memory care beds. A lot of patients without needed insurance for post-acute care needs, including nursing home placement.”* **Care Coordinator**: *“Yes, there are tons of places in the Twin Cities but people that have that typically what I see is that people that have state insurance, do not have reliable transportation. It’s just how it is. And most people are not comfortable riding in a medical taxi, all the way down to the cities with a stranger with their baby. Um, so it’s like yes, there are certain resources available, but are they ideal for a lot of families, no and if there are other children, there is no childcare, I mean, that just you know what I mean it just snowballs and all these other like other barriers, essentially, but yeah sorry I kind of got off topic there.”*
	**Partnering**	**Physician**: *“I talked about the homeless advocate. She has kind of initiated a release of information for lots of agencies in the area including the hospitals, including some of the mental health resources and housing resources. And so there’s a population of, somewhere between 100 and 200 people who have signed one of those. So if somebody has one of those signed, I feel a little bit more comfortable calling different folks and kind of connecting.”* **Case Manager**: *“There just isn’t a point person at [the] public health [department] that can help be that navigator and have that continuity with, with the organization and the community.”*
**External/State**	**Payer Policies**	**Administrator**: *“If we’re going to really drive the program well we need dedicated resources which cost money, and there’s not a really a clear financial return yet and we see we’re doing the right thing for the community but we’re paying money to keep people out of the facility which is also having an impact financially so the methodology for reimbursement in healthcare is not exactly aligned for prevention and public health and improvement in overall wellness so that’s challenging*.” ***Care Coordinator****: “So [a] TCM call is basically transitional care management - so any individual who’s discharged from a hospital setting, we follow up on from a care coordination standpoint, offering support, ensuring that, you know if they had orders for home care that they are set up and in place. Basically, our goal is to ensure that when they’re at home, they’re successful and don’t end up back in our [emergency room] or hospitalized for the same purpose and so offering to reconcile the medications, and just reviewing what their home life is like and any challenges [or] barriers, things along those lines that might play a role in a potential readmission.”*
	**Misaligned Incentives**	**Collaborative Staff**: *“Quality measures superficial – especially at hospital level – was meal nice, room,* etc. *Need to pay attention to quality measures to capture SDOH. Did the care coordinator help you get housing and transportation? Lots of work goes into that, but not measured currently.”*

#### Organization-level

At the organization-level, two subthemes were identified: work-flow and buy-in.

##### Work-flows for screening and referral

Participants described a variety of ways in which they or their organizations engaged in activities related to addressing SDOH. For example, administrators spoke of providing internal leadership to activities to address SDOH while clinicians and case managers spoke about screening and referral activities. The most frequently mentioned activities included screening for social health needs in both inpatient and outpatient settings, internal referrals (e.g. case management to care coordination), and providing resources about various community and government-based social services. Formal referrals to external organizations that can help with social health needs, actively partnering with external organizations, and providing leadership to these various efforts in the community were mentioned less frequently and indicative of them occurring less frequently.

#### Workflows as a barrier

Across these activities, several barriers were noted. Many participants pointed out that there is no systematic screening process for identifying or addressing patients’ SDOH. Across the collaborative screening occurs formally and informally in both inpatient and outpatient settings, at different times, and by different clinicians. Interviewees also described a variety of formal and informal processes for referrals made within and outside of their organizations, as well as a variety of coordination and communication processes across people and settings. In addition, there were varied processes for documenting assessment findings. Documentation inconsistencies were related to the use of different EHRs in different organizations and various frustrations with their own HCO’s EHR. Once information and referrals have been provided, there is no systematic follow-up to document whether referral services were received by patients, particularly when referral was made to community-based organizations. Lastly, there were not clear and systematic processes to advocate internally and externally the value of addressing social health needs by HCOs. A Director of Case Management described the lack of standardization internally: “*I think lack of standardization can be a challenge. And yes, overlap sometimes where the right hand doesn’t feel like it’s talking to the left hand. Yeah. And I think we’re doing a lot of work here to try and streamline what we’re doing internally as far as handoff and then to external organizations as well*.”

Some interviewees mentioned that across Wilderness Health members, and even within individual organizations, there are a multitude of different job titles, roles, and duties related to SDOH. These pose challenges for communicating and referral processes. Further, it was noted that many projects related to addressing social health needs seemed based on individual employee “champions” rather than systematically approached.

#### Work-flows as a facilitator

Participants discussed specific processes developed within their HCOs or communities that helped them address SDOH. Similarly, being smaller in size sometimes provided the ability to innovate and participants described initiatives to serve specific patient needs or to address specific challenges within their communities. Innovations mentioned included providing food vouchers to food insecure families, constructing community walking trails, and giving 211 information cards (211 is a telephone number providing information and referral to local health and human services within a community) to all patients being discharged. Some described having resource lists, scripts, and other ways of systematically communicating about resources within a given community. They also mentioned organizational or professional membership organization screening and assessment tools, evidence-based guidelines and checklist for specific conditions and potential social health needs were helpful, as well as population-level data on the community.

##### Buy-in

Participants reported a variety of perceived levels of buy-in from executives and clinicians within their organizations. Additionally, a few reported the challenge of getting every clinician on board with systematic screening and referral efforts. One physician offered this perspective on the issue*; “I think physician buy-in is huge and how do you get everybody on the same page. To support this. And again, I think part of it is a systems approach to be a dictator are meant to stress the importance of it so everybody’s doing the same thing across the system.”* At the same time, participants noted increasing and more widespread buy-in to the concepts of SDOH and value-based care more broadly (within society and among “people in health care”).

#### Community-level

At the community-level, two subthemes were identified: resources within the community and partner capacity.

##### Resources within the community

Participants mentioned a wide variety of collaborators and potential collaborators across a wide spectrum of social, health, and safety services (Indian Health Services, Veteran’s Administration, public health departments, police departments, remote monitoring companies, elder circles and volunteer transportation) and spoke highly of the people working in social services.

At the same time, participants identified numerous specific resources that were in shortage, which hindered their ability to address SDOH: mental health clinicians; transportation; Internet; long-term care including memory care, respite, personal care services and hospice; specialty care; emergency medical services; public health department staffing; general workforce; and housing, specifically Section 8 housing. Such shortages were exacerbated by the rural nature of the community, facets of which included fewer people in the labor pool available to fill positions and the distance between communities. They also mentioned how various policies and practices adversely impacted these resources. Examples included how transportation assistance is often staffed by volunteers, the legal and regulatory hurdles with providing it themselves, and Section 8 housing waiting lists. The intersection between resources and payer policies is described more below.

##### Partner capacity

Going beyond whether a particular resource exists in the community, participants also talked about whether those resources had the knowledge, time, skills, and/or resources to be reliable partners. And while some individuals mentioned specific service areas or communities where resources appear well-established and where there are high levels of engagement, more often participants discussed concerns as to whether potential social service partners in their communities had the capacity to do more. This was especially noted in areas of lower socioeconomic status and less densely populated rural areas. As one administrator described: *“They [a few partners] just don’t have resources either or maybe other challenges so I think there’s times we think we wish this group would come, engage more with us and they don’t always do that, probably good reason for it, sure.”* Another capacity issue was information sharing. Underlying this concern was the ability of partners to handle protected health information as well as the technical capabilities for sharing information. Last, participants described how their own HCOs had low capacity for partnering externally, or even just participating with community-based organizations and coalitions – these barriers were related to time and staffing barriers (e.g. not having staff time to partner).

Partnering among organizations cannot be separated from the context of the community that those organizations are in, and, as it related to the rural nature of the area, participants felt that there were high levels of community trust in which to build relationships. One social worker shared; *“I’ve worked with half of the people that I communicate with within these agencies, at a different capacity [they] are our families, they’re friends we’ve grown up together, you know, just the community itself, but yeah those exterior resources are extremely easy to get in touch with, and follow through and you know I feel confident that they’re gonna follow through where you don’t have to constantly check in, but usually it’s the same day.”*

Some participants also talked about having established agreements between partners for data sharing, the hope and promise of an electronic referral platform, and being able to leverage grants to address social health needs. Interestingly, a positive side effect of COVID-19 that was noted was more engagement with community partners on behalf of vulnerable patients. At the same time, others expressed being challenged by the time and energy required to stay aware of every service offered by every agency.

#### External/state-level

At the external/state-level, two subthemes were identified: payer policies and misaligned incentives.

##### Payer policies

Participants discussed a myriad of ways in which payers posed challenges to their own organizations beyond how such policies constrained resources. Of particular concern was reimbursement, which led to some HCOs making the investment in SDOH activities without necessarily being directly reimbursed for those services. Other payment barriers ranged from the specific (e.g. no relative value unit for providing SDOH care), to the general (e.g. Medicare does not pay for a lot of services, mismatch between what you can bill for and what patient needs), to the health system level (e.g. no system level approach to payment, still moving from fee-for-service to value-based care). Some pointed out that the money used to address SDOH came from grant money which was less sustainable. Others described how the rural demographics of elderly and low-income and the corresponding reliance on public payers was challenging. For those in leadership positions the challenges with payment made it more difficult to make the business case to add services related to addressing SDOH. For example, rural areas may not have the population to make a particular service sustainable, or cost-effective; alternatively, a person’s closest hospital may not be in the county in which they reside. As a Director of Nursing described it “*some resources are county-based and the counties are large/misshaped and for some people the largest city/town that is closest is in another county, so they tend to go there for care, when the way the funding is they are supposed to travel further, within county*.”

At the same time, specific programs which incentivize addressing SDOH were identified. It should be noted that while participants seemed to at least consider SDOH regularly in their work, it is not necessarily labelled as such. In different settings and under different payer requirements, addressing SDOH “looks different” or may be integrated into other processes. The following programs were all mentioned as accounting for a patient’s SDOH to some extent: Medicare’s annual wellness visit, and Chronic Care Management programs, transitional care management for hospital discharge, Wilderness Health’s value-based contract with Medicaid for behavioral health, and private efforts that pool rural populations or risk-adjust, or pay for screening and care management. Although all programs mentioned incorporate some level of addressing SDOH, the amount and directness of the incentives to do so vary. For example, a less direct incentive to address SDOH would be collecting information about social needs to bill for a specific process (e.g. Medicare’s chronic care management), which allows HCOs to consider SDOH more in their work but not necessarily receive more payment for addressing SDOH. Whereas, an example of a more direct incentive to address SDOH is paying more for a more socially complex population (e.g. risk adjustment). In addition, the increased use of telehealth and payment parity, in part related to the COVID-19 pandemic, were viewed as promising.

##### Misaligned incentives

In contrast to the specific payer policies that impact the organizations comprising Wilderness Health, participants also spoke about the fact that potential partners from organizations outside the specific one they work in don’t have the same set of payment incentives when it comes to addressing SDOH, or even for providing more value-based care. Within the Wilderness Health collaborative and the surrounding communities each is located in there is a mix of different organizations serving health and social service needs - some are for-profit, others are federally qualified health centers, others still are community-based volunteer organizations - and each comes with its own set of payment mechanisms. These different payment mechanisms vary in the level and directness too and thereby affect an organization’s ability to partner well and address SDOH effectively. One example was how critical access hospitals receive cost plus reimbursement for hospitalizations, while also being under separate ownership from the primary care facilities in their area, so SDOH in the community is not prioritized. One administrator shared; “*So [a] good example is in a community where you have a [critical access] hospital and a federally qualified health center or clinic that’s separate. They’re interdependent in a way but they’re getting paid different, they’re not aligned and so for them there’s like probably no incentive to do it different if they’re doing well under grant funding the funding keeps coming in, so why change.”*

A few participants mentioned that there are no quality metrics from the state or public payers related to SDOH such as whether the care coordinator helped connect a person with housing/transportation. This was important as HCOs are motivated to meet certain state metrics and felt that metrics related to addressing and impacting SDOH would help motivate the importance of the work.

## Discussion

Numerous barriers affected the ability of rural health care organizations to address SDOH. These barriers reflected both general challenges to health care delivery in the U.S. (e.g. high cost, multi-payer, insurance-based system) and those specific to addressing SDOH. Process barriers existed within individual organizations and within the collaborative as a whole, with some of these process barriers seeming to be adaptations/responses to the external barriers (e.g. processes to fit payer demands vs. a comprehensive approach to SDOH). Alignment of incentives between payers, HCOs, and social service agencies may help drive the work to address SDOH and may also lead to developing more robust internal processes and structural capacity. Additionally, bolstering internal capacity to improve workflow around SDOH holds potential, such as standardization of screening, shared accountability, and incorporation into the EHR. As others have found, it’s not a lack of support but lack of time and resources [23].

This study’s findings echo what others have found nationally and in larger, more integrated health systems, including ACOs [10–13, 24] and speak to the need to build partnership capacity [25]. Findings point to several ways in which more loosely affiliated HCOs could help further the work of addressing SDOH. The first is to develop overarching processes for screening and referral, standardize screening tools and implement them more systematically, across organizations. This is particularly true in outpatient settings, where there are fewer connections between SDOH activities and payment. Then, once screening tools are in place there needs to be additional steps to improve screening rate and connecting patients to resources in order to address patient health needs [26]. Processes should also be able to be tailored to the nuances of members’ organizational and affiliated facilities. Collaboratives serve as conveners, and thus have the ability to share innovations and evidenced-based guidelines across members and help develop resources lists for communities. Alternatively, a referral platform could be purchased and utilized, but ideally would need to be interoperable across the variety of EHRs. Collaboratives should continue to identify and pursue value-based care opportunities and work towards strategic alignment of incentives across different organizations [12]. Collaboratives can also advocate for increasing social services investment [27] and foster data sharing [12] as viable ways to increase the capacity of the social service sector to engage with health care. Similarly, advocacy efforts to expand permanent telehealth payment parity should be undertaken [28]. Last, there should be efforts to develop appropriate quality metrics related to SDOH processes and outcomes. Additionally, collaboratives could consider direct investment for social health needs or directly purchasing specific services in communities [2]. These findings and the corresponding policy/practice implications are especially important given how socioeconomic factors contribute to rural health disparities [29].

The findings need to be considered in the context of the unique challenges rural HCOs face in providing health care to their populations. Chen and colleagues [10] bring up the importance of greater market share as a facilitator. Although rural HCOs may appear to have “full market share” because there are no other options, they don’t have enough volume to leverage payers and there may be mixed ownership (e.g. the clinic and hospital have different owners), so incentives in the community are misaligned. Our findings are consistent with Murray et al. [12] who note that small collaborations had similar challenges to larger organizations, but cited a need for data sharing and better capabilities of partners. Among our participants, being rural meant an increased reliance on relationships to get things done, so partnership capacity may be even more important. Developing partnerships holds great potential as dense multi-sectoral partnerships have had demonstrated positive health effects [30]. Moreover, rural communities may be well positioned for dense multi-sector partnering given their smaller scale and the interlocking positions held by community leaders (meaning those in leadership roles within health care may also serve in leadership positions in community or social service agencies). At the same time, even within mature partnerships, most groups lacked the power to transform the current financing structures within regional health systems and lacked coordination and the ability to share data [25], so partnering cannot be a substitute for policy change.

More research is needed on the capacity-building, technology and resources needs [31]. This is particularly true in rural areas. Likewise, more research is needed on the perspectives of social service partners. Past research indicates the need for strong relationships, going beyond just referral networks [32]. State legislatures and community foundations/funders should consider enhanced investment in non-profits and social services organizations that may receive increased referrals from HCOs. Recently, stakeholders of rural health in Minnesota convened and identified investments in social determinants of health will be a major disrupter of how care is delivered [33]. It also comes in the midst of the COVID-19 pandemic, which has dramatically and rapidly increased social health needs across the country [34].

### Limitations

Although this study’s findings align with what other researchers have found in larger health systems, Medicare ACOs, and among rural stakeholders within Minnesota, there are limitations in extending this research to other rural communities. This study focused on one collaborative in rural northern Minnesota and western Wisconsin. Even within Minnesota rural communities are unique from one another. However, the fragmentation of payer policies, misalignment between state and federal initiatives and HCO financial viability is not unique to this study. Another limitation is that participants were selected to participate in interviews given their position and ongoing work with SDOH activities. While this allowed for rich description of activities and the corresponding challenges and barriers, their views may not represent the views of others within the collaborative. At the same time, their perspectives were similar to those in other settings, given the agreement between our findings and those of others.

## Conclusions

Rural providers have unique needs when it comes to addressing social health needs. Collaboration among rural health systems should be fostered to develop common protocols, promote value-based care, and offer economies of scale to leverage value-based payment. Changing policy is required to help rural providers be viable, and better align incentives across health care entities. Further, states can help align incentives and performance metrics across various rural health care entities, engage payers in promoting and paying for value-based care, and in bolstering social service capacity. As our health care system evolves to meet population health needs, policy needs to evolve to support coordination among rural health care providers and promote support from local social services.

## Data Availability

All data generated from this study is available from the corresponding author on reasonable request.
